# Alleviation of Hepatic Steatosis: Dithizone-Related Gut Microbiome Restoration During Paneth Cell Dysfunction

**DOI:** 10.3389/fmicb.2022.813783

**Published:** 2022-02-25

**Authors:** Saisai Zhang, Hein M. Tun, Dengwei Zhang, Hau-Tak Chau, Fung-Yu Huang, Hin Kwok, Danny Ka-Ho Wong, Lung-Yi Mak, Man-Fung Yuen, Wai-Kay Seto

**Affiliations:** ^1^Department of Medicine, The University of Hong Kong, Hong Kong, Hong Kong SAR, China; ^2^HKU-Pasteur Research Pole, School of Public Health, The University of Hong Kong, Hong Kong, Hong Kong SAR, China; ^3^School of Public Health, Nanjing Medical University, Nanjing, China; ^4^Centre for PanorOmic Sciences, LKS Faculty of Medicine, The University of Hong Kong, Hong Kong, Hong Kong SAR, China; ^5^State Key Laboratory of Liver Research, The University of Hong Kong, Hong Kong, Hong Kong SAR, China; ^6^Department of Medicine, The University of Hong Kong-Shenzhen Hospital, Shenzhen, China

**Keywords:** NAFLD, MAFLD, microbiota, *Bacteroides*, metabolic, steatosis

## Abstract

Non-alcoholic fatty liver disease (NAFLD), the world’s most common chronic liver disease, is increasingly linked to gut dysbiosis. Paneth cells secrete antimicrobial peptides that regulate the gut microbiome, but their role in the pathogenesis of NAFLD remains unclear. Here, we determine the changes in NAFLD development and gut microbial composition and function *via* the injection of dithizone that can pharmacologically deplete the granules of Paneth cells. Eight-week-old C57BL/6J male mice (*n* = 31) were given a high-fat diet (HFD) or standard control diet for 12 weeks. Dithizone (10 mg/kg) was intravenously injected every 3 weeks during the period of diet feeding. Metagenomic DNA was extracted from fecal samples for PacBio Single-Molecule Real-Time sequencing to identify changes in microbial composition and predicted function. We observed dithizone-treated HFD mice, when compared to non-treated HFD mice, to have significant reductions in hepatic triglyceride content (28.98 vs. 53.52 mg/g, *p* = 0.0419); plasma insulin level (2.18 vs. 6.63 ng/ml, *p* = 0.0079); and relative mRNA levels of fatty acid synthase (0.52 vs. 1.57, *p* = 0.0428) and stearoyl-CoA desaturase-1 (0.43 vs. 1.20, *p* = 0.0121). Bacterial taxonomic profiling found dithizone-treated HFD mice, when compared to non-treated HFD mice, had a lower *Firmicutes/Bacteroidetes* ratio (2.53 vs. 5.26, *p* = 0.0541); a higher relative abundance of *Bacteroides ASV21* and *ASV42* (1.04 vs. 0.22%, *p* = 0.0277 and 0.96 vs. 0.09%, *p* = 0.0213); and a reduction in microbes belonging to *Firmicutes* (all *p* < 0.05). *Bacteroides* species correlated positively with predicted microbial functions such as L-methionine (*r* = 0.54, *p* = 0.0019) and tetrahydrofolate (*r* = 0.52, *p* = 0.0029) biosynthesis. Collectively, dithizone treatment was associated with alleviation in the severity of liver steatosis in HFD mice, possibly through gut microbiome modulation involving the increase in *Bacteroides*, suggesting microbiome-targeted therapies may have a role in the treatment of NAFLD.

## Introduction

The general population with non-alcoholic fatty liver disease (NAFLD) is increasing owing to the prevalence of obesity and metabolic risk factors (Bedogni et al., 2005; Cohen et al., 2011). There is emerging evidence demonstrating the gut microbiome as a critical environmental factor that affects the onset and progression of NAFLD. Germ-free mice with a high-fat diet (HFD) were found to gain less weight than conventional mice, with weight gain replenished by microbiota transplantation (Backhed et al., 2004, 2007). The involvement of the gut microbiome may contribute to the progression from simple hepatic steatosis to liver fibrosis and cirrhosis (Henao-Mejia et al., 2012; Le Roy et al., 2013; Mouzaki et al., 2013; Boursier et al., 2016). Based on 16S rRNA gene sequencing, obese NAFLD patients showed an increase in the levels of *Firmicutes Lactobacillus* and a reduction in the levels of *Firmicutes Oscillibacter* when compared to healthy controls (Michail et al., 2015).

Many efforts were put on to explain how gut microbiome dysbiosis leads to NAFLD development. The increased intestinal permeability caused by gut microbiome dysbiosis promotes translocation of metabolites into portal blood circulation. Responses to this dysbiosis promote liver damage (Miele et al., 2009; Bashiardes et al., 2016; Poeta et al., 2017). However, the exact role of the microbiome and its interaction with the pathogenesis of NAFLD remains undetermined. Exploring underlying regulatory mechanisms may contribute a deeper understanding of NAFLD and on the potential of microbiome-based interventions.

The specialized epithelial cells in the small intestine, named Paneth cells, play a homeostatic role in establishing and maintaining the intestinal microbiota by secreting antimicrobial peptides and proteins (Wilson et al., 1999; Bevins and Salzman, 2011). A previous study indicated that the percentage of *Firmicutes* was significantly lower and the percentage of *Bacteroidetes* was significantly higher in defensin-overexpressing mice, with the opposite observed in defensin-deficient mice (Raman et al., 2013; Jiang et al., 2015). These findings directly demonstrated that alterations in Paneth cell defensin expression have a significant impact on the bacterial composition of the microbiota. Given the regulatory role of Paneth cells in the microbial microenvironment, they are largely investigated in irritable bowel syndrome and Crohn’s disease (Stappenbeck and McGovern, 2017; Zhou et al., 2020). Paneth cells may be associated with other metabolic disorders, but there is a paucity of data about their role in the pathogenesis of NAFLD. We hence aimed to determine whether Paneth cells are involved in the development of NAFLD by injection of dithizone, which can disrupt cell granulates (Sawada et al., 1991; Lueschow et al., 2018; Berger et al., 2019; Guo et al., 2019; Liu et al., 2019), and identify Paneth-cell-oriented microbial alterations.

## Materials and Methods

### Mouse Model

Eight-week-old male C57BL/6J mice (*n* = 31, Center of Comparative Medicine Research, The University of Hong Kong) were given an HFD (containing 60% fat from lard, 20% carbohydrates, and 20% proteins, D12492, Research Diets, New Brunswick, NJ) or a standard control diet (STD, containing 10% fat from lard, 70% carbohydrates, and 20% proteins, D12450J, Research Diets, New Brunswick, NJ) for 12 weeks to establish a NAFLD mouse model. The granulates in Paneth cells were depleted pharmacologically by intravenous injection of dithizone (Sawada et al., 1991; Lueschow et al., 2018; Berger et al., 2019; Guo et al., 2019; Liu et al., 2019) (10 mg/kg body weight, dissolved in 10 mg/ml lithium carbonate solution, Sigma-Aldrich, Darmstadt, Germany) every 3 weeks during the period of diet feeding. Mice were randomly divided into four groups (*n* = 7–8): (i) mice fed with STD and injected with the vehicle (SC group); (ii) mice fed with STD and injected with dithizone (SD group); (iii) mice fed with HFD and injected with the vehicle (HC group); and (iv) mice fed with HFD and injected with dithizone (HD group). All mice were maintained under controlled environmental conditions (23 ± 1°C, 50–60% humidity, 12-h light/dark cycles) with food and water *ad libitum*. Body weight was monitored weekly. All animal experimental procedures were approved by the Committee on the Use of Live Animals in Teaching and Research at the University of Hong Kong (reference number: CULATR 4507-17) and received humane care according to the criteria outlined in the “Guide for the Care and Use of Laboratory Animals.”

### Glucose Tolerance and Insulin Tolerance Tests

After a 12-week intervention, glucose tolerance tests were performed in all mice injected with D-glucose (2 g/kg body weight) intraperitoneally after being fasted for 16 h (overnight, between 6 p.m. and 10 a.m.). For insulin tolerance tests, all mice were intraperitoneally injected with insulin (0.75 IU/kg body weight) after being fasted for 6 h (between 10 a.m. and 4 p.m.). Blood glucose levels were monitored at various time points (0, 15, 30, 60, and 120 min) with an ACCU-CHEK Glucose Meter (Roche Diabetes Care, Inc., Basel, Switzerland).

### *In vivo* Intestinal Permeability Test

Mice were fasted for 6 h and orally administrated with fluorescein isothiocyanate (FITC)-dextran (4 kDa, 44 mg/kg bodyweight, Sigma-Aldrich, Darmstadt, Germany). After a 1-h treatment, whole blood was collected from the tail vein and centrifuged at 3,000 rpm for 15 min. FITC-dextran was diluted with 1× phosphate buffer saline into 0, 27.5, 55, 110, 220, 440, 880, and 1,760 ng/ml to make a standard curve, and plasma samples were also diluted to a 1:10 ratio. Plasma FITC-dextran concentrations were measured at an excitation wavelength of 485 nm and an emission wavelength of 535 nm as previously reported (Fernandez-Carrera et al., 2017).

### Biochemical Measurement in Plasma Samples

After a 12-week intervention and all *in vivo* tests, all mice were sacrificed under anesthesia. Whole-blood samples were collected by cardiac puncture and centrifuged at 3,000 rpm for 15 min. The circulating Paneth cell-specific α-defensin 5 (DEFA5) levels (Abbexa Ltd., Cambridge, United Kingdom) and insulin levels (Antibody and Immunoassay Services, HKU, Hong Kong) were detected by way of enzyme-linked immunosorbent assay (ELISA) according to the manufacturer’s instructions.

### Histological and Immunofluorescence Staining

Mouse liver tissues were embedded with both optimal cutting temperature compound and paraffin to make frozen and paraffin sections. The paraffin sections were prepared in 5 mm sizes, dehydrated in graded ethanol and xylene, and then processed for hematoxylin and eosin (H&E) and Masson’s trichrome staining following standard protocols, with the frozen sections stained using Oil Red O (Sigma-Aldrich, Darmstadt, Germany). Mouse ileal sections were stained with H&E and immunostained with an anti-lysozyme antibody (Abbexa Ltd., Cambridge, United Kingdom).

### Hepatic Lipid Extraction and Measurement

The lipids were extracted from liver tissues using the chloroform–methanol method (Folch et al., 1957) and were measured with the responding triglyceride and total cholesterol kits according to the manufacturer’s instructions. The mRNA levels of fatty acid synthesis-related genes fatty acid synthase (FASN) and SCD1 and fatty acid β-oxidation-related gene CTP1a were measured by quantitative PCR (qPCR).

### Western Blot and Quantitative PCR

Proteins and RNAs were extracted from mouse livers and the ileum using a radioimmunoprecipitation assay buffer (Sigma-Aldrich, Darmstadt, Germany) and TRIzol reagent (Invitrogen). The protein concentration was determined by using the BCA protein assay (Thermo Fisher Scientific, Waltham, MA, United States) according to the manufacturer’s instructions. Anti-lysozyme rabbit antibody (Abbexa Ltd., Cambridge, United Kingdom), anti-ZO-1 rabbit antibody (Thermo Fisher Scientific, Waltham, MA, United States), anti-occludin rabbit antibody (Thermo Fisher Scientific, Waltham, MA, United States), and anti-β-actin mouse antibody (Thermo Fisher Scientific, Waltham, MA, United States) were used. The protein bands were visualized and imaged with the ChemiDoc™ MP Imaging System (Bio-Rad, Hercules, CA, United States), with the densities analyzed by ImageJ software. qPCR was performed using the DNA Engine Opticon^®^ 2 System for Real-Time PCR Detection (Bio-Rad, Hercules, CA, United States) with TB Green Premix Ex Taq (Tli RNase H Plus) (Takara, Kusatsu, Shiga, Japan) and specific primers. The relative levels of gene expressions were calculated by the 2^–Δ^ ^Δ^ *^Ct^* method, after normalization with the abundance of β-actin. The sequences of primers used in this study were listed in Supplementary Table 1.

### Paneth Cell Functions

The protein level of lysozyme, a specific marker of Paneth cell (Sato et al., 2011), was determined by immunofluorescence staining and western blot analysis. Angiogenin 4 (ANG4) and regenerating islet-derived protein 3 gamma (Reg3γ) mRNA levels, antimicrobial peptides produced by Paneth cells as part of the innate immune response, were determined by qPCR.

### Full-Length 16S Sequencing by PacBio Single-Molecule, Real-Time Technology

Stool DNAs were extracted by using a QIAamp PowerFecal DNA kit (Qiagen, Hilden, Germany) according to the manufacturer’s instructions. Standard agarose gel electrophoresis was applied to evaluate the quality of DNA samples, and DNAs were quantified using a NanoDrop 2000c spectrophotometer (Thermo Fisher Scientific, Waltham, MA, United States).

DNA library preparation was performed following the Full-Length 16S Amplification SMRTbell Template Preparation workflow. For generating tagged amplicons, first-round PCR (16S amplification) was performed using the 16S primers tailed with universal sequences (27F: 5AmMC6-gcagtcgaacatgtagctga ctcaggtcac-AGRGTTYGATYMTGGCTCAG and 1492R: 5Am MC6-tggatcacttgtgcaagcatcacatcgtag-RGYTACCTTGTTACGAC​ TT) and 2.5 ng input of gDNA with 20 PCR cycles. For generating barcoded amplicons, second-round PCR (barcoding amplification) for multiplexing reactions was performed using PacBio barcoded universal primers and 2 ng of tagged amplicons as input with nine PCR cycles. The KAPA HiFi Hot Start DNA Polymerase (KAPA Biosystems) was used to perform with denaturing at 95°C for 30 s, annealing at 57°C for 30 s, and extension at 72°C for 60 s. Post-amplification quality control was performed on a Bioanalyzer (Agilent Technologies, Santa Clara, CA, United States). SMRTbell library construction was performed by pooling the barcoded amplicons in equimolar concentrations, followed by DNA damage repair, SMRTbell ligation, and purification (SMRTbell library). Sequencing template preparation of the SMRTbell library was then performed using the binding calculator as instructed by PacBio followed by sequencing on the PacBio Sequel System.

### Microbiome Analysis

Prior to the analysis, subreads were converted from raw PacBio sequencing data to highly accurate Q30 circular consensus sequencing (CCS) reads using Single-Molecule, Real-Time (SMRT) Link 3.1.1 software. Sequences were filtered with four full passes and a minimum predicted accuracy of 0.999. The DADA2 method (Callahan et al., 2016) was used in these processing steps: filtering, dereplication, denoising, amplicon sequence variant (ASV) inference, chimera, and merging. Taxonomy assignment was based on the naive Bayesian classifier with the SILVA v132 database (Quast et al., 2013) and BLAST searches against the NCBI nucleotide database (nt), at the genus level and species level, respectively. A minimum subsampling depth of 4,556 reads was selected for microbiota composition analysis.

Chao 1 (a measure of community richness) and Shannon’s index (a measure of richness and evenness) were used to describe the alpha diversity of microbial community. For beta diversity, unweighted UniFrac distance (a qualitative measure of community dissimilarity) was adopted. Principal coordinate analysis (PCoA) was performed using the vegan package^1^ of R (Version 3.4.4). The comparison of taxa between groups was tested by the Wilcoxon rank sum test when the taxa were present in more than 50% of samples; otherwise, Fisher’s exact test was adopted based on the presence/absence of taxa. The *p*-value was corrected using the Benjamini–Hochberg method (Benjamini and Hochberg, 1995), which controlled the false discovery rate (FDR). The log2-fold change in the relative abundance of individual ASVs was calculated manually with log2(abundance in case group + 1)/(abundance in control group + 1). ASVs were separated horizontally by genus and colored by phylum. Spearman’s rank correlation coefficient was adopted to understand the correlation, and the co-occurrence network was visualized with the Cytoscape software (Shannon et al., 2003) if |Spearman’s rank correlation coefficient| > 0.3 and adjusted *p*-value < 0.05. For pathway prediction, PICRUSt2 (Douglas et al., 2020) was employed for functional annotation to the MetaCyc database (SRI International, Menlo Park, CA, United States). Significantly differential pathways were calculated and visualized by STAMP (Parks et al., 2014) with the extended error bar, indicating statistically significant features along with the *p*-values, effect sizes, and confidence intervals. The generated sequencing data were deposited in the European Nucleotide Archive database with the accession number PRJEB46885.

### Statistical Analysis

All data were analyzed using GraphPad Prism 7.0 (GraphPad Software, San Diego, CA, United States) or R (Version 3.4.4) and presented as the mean ± standard error of the mean. Homeostatic Model Assessment for Insulin Resistance (HOMA-IR) was calculated according to the following formula (Matthews et al., 1985): [fasting insulin (μU/ml) × fasting glucose (mmol/L)]/22.5. To examine the significant differences between every two groups, the data were compared using unpaired two-tailed Student’s *t*-test as appropriate. A *p*-value < 0.05 was considered statistically significant.

## Results

### Presence of Paneth Cell Dysfunction in Mice With Dithizone Treatment or High-Fat Diet Feeding

**FIGURE 1:**
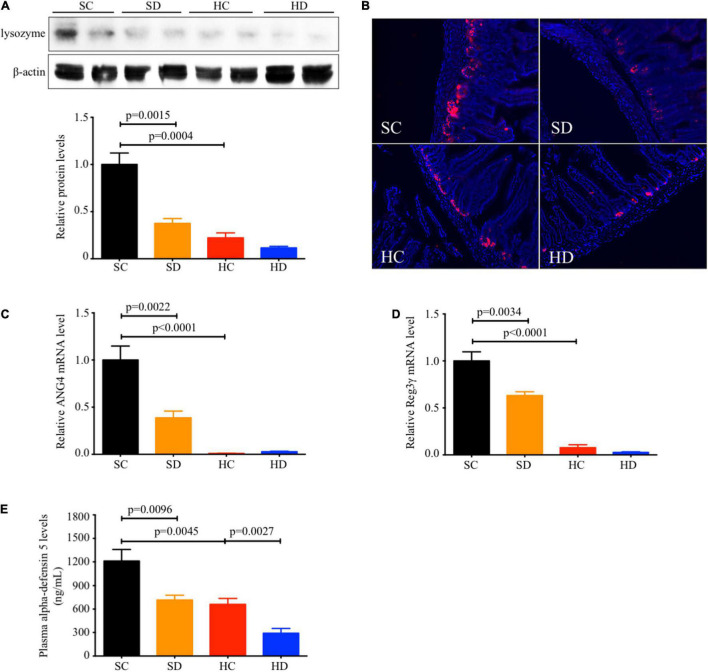
Both HFD and dithizone resulted in Paneth cell dysfunction. Eight-week-old male C57BL/6J mice were fed with STD or HFD for 12 weeks. The mice were intravenously injected with either vehicle or dithizone (10 mg/kg) every 3 weeks for a 12-week diet in total (*n* = 7–8). **(A)** The protein expression of lysozyme (a marker of Paneth cells) in the ileum was determined by western blot analysis and densitometric quantification of lysozyme. **(B)** Representative images of immunofluorescent staining of lysozyme with ×400 magnification. **(C,D)** The relative mRNA levels of ANG4 and Reg3γ. **(E)** Plasma alpha-defensin five levels were assayed by ELISA. Data are expressed as mean ± SEM for each group with unpaired Student’s *t*-test. SC, standard control diet/control; SD, standard control diet/dithizone; HC, high-fat diet/control; HD, high-fat diet/dithizone; ANG4, angiogenin 4; Reg3γ, regenerating islet-derived protein 3 gamma; ELISA, enzyme-linked immunosorbent assay; SEM, standard error of the mean.

In STD mice, the ileal protein expression of lysozyme (mean: 0.38 ± 0.05 vs. 1.00 ± 0.12, *p* = 0.0015, Figures 1A,B), the relative mRNA levels of antimicrobial peptides ANG4 (mean: 0.39 ± 0.07 vs. 1.00 ± 0.15, *p* = 0.0022, Figure 1C), Reg3γ (mean: 0.63 ± 0.04 vs. 1.00 ± 0.10, *p* = 0.0034, Figure 1D), and the plasma alpha-defensin 5 level (mean: 716.2 ± 60.71 vs. 1,211 ± 148.8 ng/ml, *p* = 0.0096, Figure 1E) were significantly reduced in dithizone-treated mice when compared to non-treated mice. Similar results were also observed between non-treated STD and non-treated HFD mice (all *p* < 0.05, Figures 1A–E). No significant differences were noted between non-treated HFD and dithizone-treated HFD mice (all *p* > 0.05) except alpha-defensin 5, one of the major antimicrobial peptides. Dithizone-treated HFD mice had a significantly lower plasma alpha-defensin 5 level than non-treated HFD mice (mean: 290.4 ± 62.28 vs. 660.4 ± 75.88 ng/ml vs. *p* = 0.0027, Figure 1E).

### The Inhibitory Effects of Dithizone on High-Fat Diet-Induced Hepatic Lipid Accumulation

**FIGURE 2:**
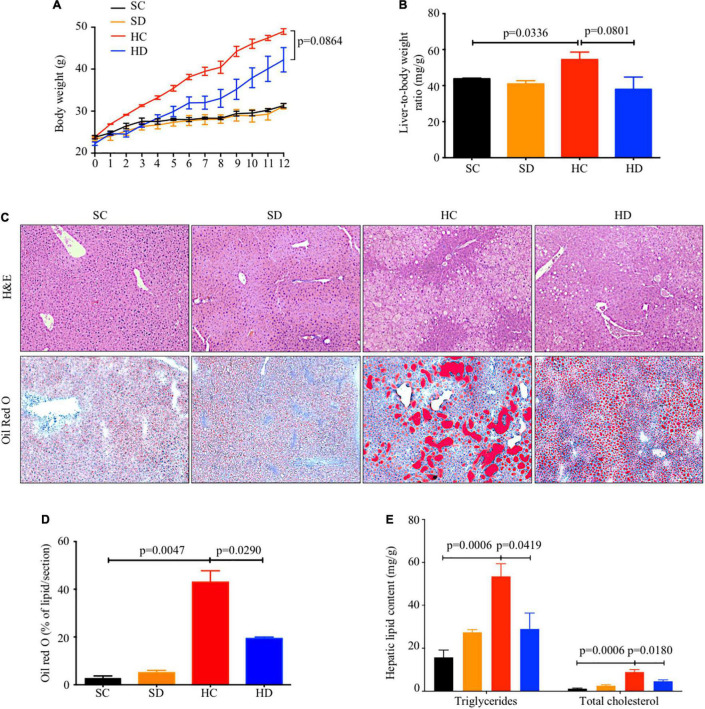
The inhibitory effects of dithizone on HFD-induced lipid accumulation. Eight-week-old male C57BL/6J mice were fed with STD or HFD for 12 weeks. The mice were intravenously injected with either vehicle or dithizone (10 mg/kg) every 3 weeks for a 12-week diet in total (*n* = 7–8). **(A)** Dynamic changes in body weight during the 12-week intervention. **(B)** Liver-to-body weight ratio after the 12-week intervention. **(C)** Representative images of hematoxylin and eosin (upper) and Oil Red O staining (lower) in the liver sections with ×100 magnification. **(D)** Quantification of Oil Red O-stained area using ImageJ software. **(E)** Intrahepatic triglyceride and total cholesterol content. Data are expressed as mean ± SEM for each group with unpaired Student’s *t*-test. SC, standard control diet/control; SD, standard control diet/dithizone; HC, high-fat diet/control; HD, high-fat diet/dithizone; H&E, hematoxylin and eosin; ORO, Oil Red O; SEM, standard error of the mean.

Both body weight (Figure 2A) and liver-to-body weight ratio (Figure 2B) were significantly increased by HFD feeding (mean: 31.30 ± 0.51 vs. 48.95 ± 0.70 g, *p* < 0.0001; 43.86 ± 0.30 vs. 54.48 ± 4.14 mg/g, *p* = 0.0336) and partially reversed with borderline significance by dithizone treatment in HFD mice (mean: 48.95 ± 0.70 vs. 42.21 ± 2.90 g, *p* = 0.0864; 54.48 ± 4.14 vs. 37.98 ± 6.78 mg/g, *p* = 0.0801). Based on H&E staining and Oil Red O staining, HFD-induced hepatic lipid accumulation was significantly decreased in dithizone-treated HFD mice compared with non-treated HFD mice (19.38 ± 0.59% vs. 42.99 ± 4.72%, *p* = 0.0290, Figures 2C,D). Similarly, dithizone-treated HFD mice had a significant reduction in mean intrahepatic triglyceride (28.98 ± 7.40 vs. 53.52 ± 5.93 mg/g, *p* = 0.0419) and total cholesterol content (4.62 ± 0.68 vs. 8.96 ± 1.16 mg/g, *p* = 0.018) when compared to non-treated HFD mice (Figure 2E). No aforementioned significant differences were noted between non-treated and dithizone-treated STD mice.

The relative mRNA levels of fatty acid synthesis-related genes FASN and stearoyl-CoA desaturase-1 (SCD1) were significantly downregulated in dithizone-treated HFD mice when compared to non-treated HFD mice (0.52 ± 0.18 vs. 1.57 ± 0.43, *p* = 0.0428; 0.43 ± 0.12 vs. 1.20 ± 0.23, *p* = 0.0121), whereas fatty acid β-oxidation-related gene carnitine palmitoyltransferase 1a (CTP1a) was upregulated (1.67 ± 0.27 vs. 1.05 ± 0.14, *p* = 0.0653, Supplementary Figure 1).

### Dithizone Improved High-Fat Diet-Induced Glucose Intolerance and Insulin Resistance

**FIGURE 3:**
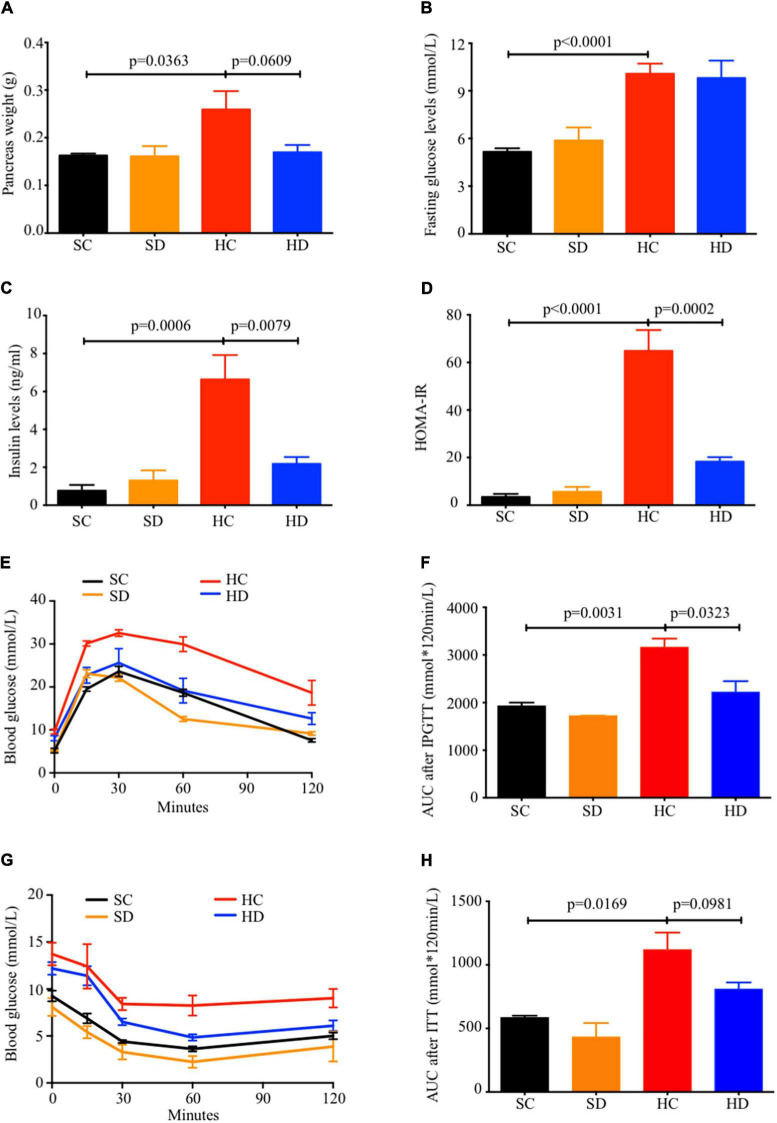
Dithizone ameliorates HFD-induced glucose intolerance and insulin resistance. Eight-week-old male C57BL/6J mice were fed with STD or HFD for 12 weeks. The mice were intravenously injected with either vehicle or dithizone (10 mg/kg) every 3 weeks for a 12-week diet in total (*n* = 7–8). **(A)** Pancreas weight after the 12-week intervention. **(B)** Fasting blood glucose level after the 12-week intervention. **(C)** Fasting insulin levels after the 12-week intervention. **(D)** HOMA-IR was calculated according to the following formula: [fasting insulin (μU/L) × fasting glucose (nmol/L)]/22.5. **(E,F)** Glucose tolerance was determined by an intraperitoneal glucose tolerance test (IPGTT), and the total area under the curve (AUC) for the 0–120 min period was calculated. **(G,H)** Insulin resistance was determined by an insulin tolerance test (ITT) and quantification of AUC. Data are expressed as mean ± SEM for each group with unpaired Student’s *t*-test. SC, standard control diet/control; SD, standard control diet/dithizone; HC, high-fat diet/control; HD, high-fat diet/dithizone; AUC, area under the curve; SEM, standard error of the mean.

Pancreatic weight was reduced in dithizone-treated HFD mice when compared to non-treated HFD mice (mean: 0.17 ± 0.02 vs. 0.26 ± 0.04 g, *p* = 0.0609, Figure 3A), while no significant differences in fasting blood glucose level was observed (Figure 3B). In addition, non-treated HFD mice exhibited progressive development of glucose intolerance, hyperinsulinemia, and insulin resistance when compared to STD mice (Figures 3C–H). This was not seen in dithizone-treated HFD mice, with significantly lower insulin levels (mean: 2.18 ± 0.36 vs. 6.63 ± 1.29 ng/ml, *p* = 0.0079, Figure 3C), HOMA-IR (mean: 18.31 ± 1.87 vs. 64.90 ± 8.71, *p* = 0.0002, Figure 3D), and glucose intolerance (mean area under curve: 2,220 ± 230.1 vs. 3,163 ± 181.0, *p* = 0.0323, Figures 3E,F) when compared to non-treated HFD mice. Insulin resistance also showed a similar decrease (mean area under curve: 809.3 ± 52.53 vs. 1,119 ± 134.4, *p* = 0.0981, Figures 3G,H). No aforementioned significant differences were observed between non-treated and dithizone-treated STD mice.

### Dithizone Treatment on Gut Permeability

An *in vivo* gut permeability test showed that the FITC-dextran concentration was significantly increased by HFD feeding in non-treated mice (715.2 ± 13.86 vs. 1,091 ± 147.6 ng/ml, *p* = 0.0239). Dithizone-treated HFD mice had a numeric reduction in FITC-dextran concentration (855.6 ± 36.46 vs. 1,091 ± 147.6 ng/ml, *p* = 0.1708) when compared to non-treated HFD mice (Supplementary Figure 2A). The expression of tight junction proteins zonula occludens (ZO)-1, a marker of intestinal permeability, was downregulated by HFD feeding (mean: 1.00 ± 0.01 vs. 0.49 ± 0.09, *p* = 0.0326), and partially restored with borderline significance by dithizone treatment (mean: 1.95 ± 0.37, *p* = 0.0604, Supplementary Figure 2B).

### Dithizone Induces Gut Microbial Shifts in High-Fat Diet Mice

**FIGURE 4:**
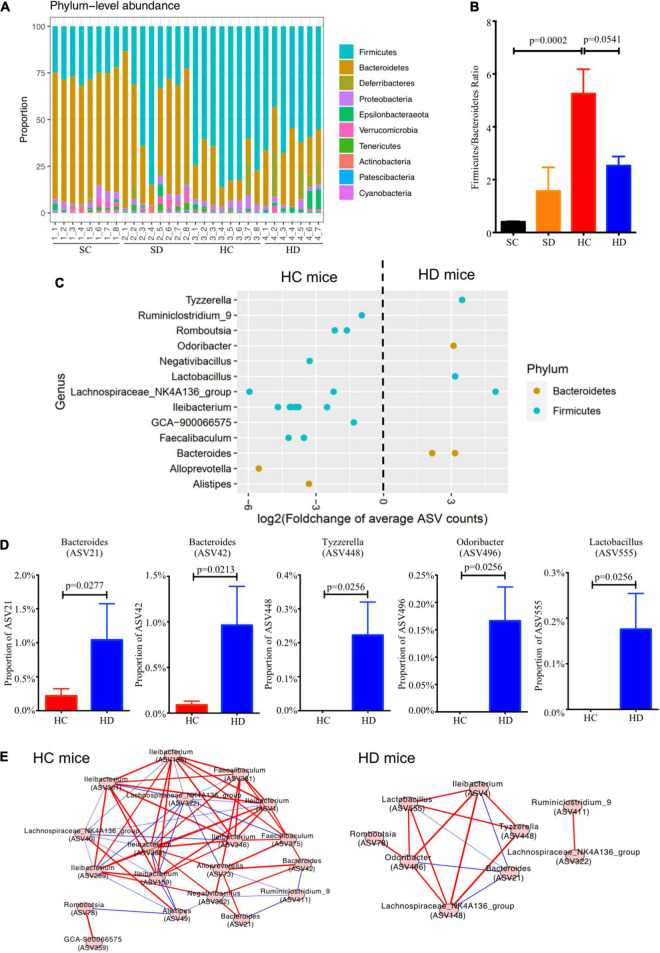
Dithizone modulates gut microbiota in HFD-fed mice. Mouse fecal microbiome from four groups were analyzed by using PacBio SMRT sequencing (*n* = 7–8 for each group). **(A)** The microbial profiles at the genus level. **(B)** The ratio of *Firmicutes* to *Bacteroidetes*. **(C)** Log2-fold change in the relative abundance of ASVs was used to identify the altered bacterial genera. ASVs were separated horizontally by genus and colored by phylum. **(D)** The proportion of altered ASVs. **(E)** The co-occurrence networks were used to understand the bacterial interaction at the genus level under different groups. Spearman’s rank correlation coefficient was adopted to understand the correlation, and the co-occurrence network was visualized if | Spearman’s rank correlation coefficient| was > 0.3 and *p*-value was < 0.05. The comparison was tested by the Wilcoxon rank sum test. The *p*-value was corrected using the Benjamini–Hochberg method. A red line indicates a positive correlation, and a blue line indicates a negative correlation. SC, standard control diet/control; SD, standard control diet/dithizone; HC, high-fat diet/control; HD, high-fat diet/dithizone; ASVs, individual amplicon sequence variants.

Altogether, 247,277 reads were obtained from PacBio SMRT sequencing. In terms of bacterial taxonomic profiling at the phylum level (Figure 4A), no considerable differences were observed in the alpha diversity indices (Chao 1 and Shannon’s index) within these four groups (Supplementary Figure 3A). PCoA based on unweighted UniFrac distance demonstrated that the gut microbiota was significantly altered in dithizone-treated STD mice when compared to those in non-treated STD mice (*p* = 0.0015, Supplementary Figure 3B). The difference was also observed between non-treated and dithizone-treated HFD mice (*p* = 0.0958, Supplementary Figure 3B). The compositions of gut microbiota in non-treated HFD mice and treated STD mice differ greatly from those in non-treated STD mice (Supplementary Figure 4). The *Firmicutes/Bacteroidetes* ratio was significantly increased by HFD feeding (mean: 5.26 ± 0.92 vs. 0.41 ± 0.02, *p* = 0.0002) and partially reversed with borderline significance by dithizone treatment (mean: 2.53 ± 0.34, *p* = 0.0541, Figure 4B).

At the genus level, *Bacteroides*, *Odoribacter*, *Lactobacillus*, and *Tyzzerella* were significantly enriched whereas *Ruminiclostridiumin_9*, *Romboutsia*, *Ileibacterium*, *Faecalibaculum*, and *GCA-900066575*, which all belong to *Firmicutes* were decreased in dithizone-treated HFD mice when compared to non-treated HFD mice (all *p* < 0.05, Figure 4C). Among them, dithizone-treated HFD mice had a significantly higher mean abundance of *Bacteroides ASV21* and *ASV42* (1.04 ± 0.54% vs. 0.22 ± 0.11%, *p* = 0.0277, and 0.96 ± 0.43% vs. 0.09 ± 0.04%, *p* = 0.0213), *Tyzzerella ASV448*, *Odoribacter ASV496*, and *Lactobacillus ASV555* (0.22 ± 0.10%, 0.17 ± 0.06%, and 0.18 ± 0.08% vs. 0.00 ± 0.005, all *p* = 0.0256) than non-treated HFD mice (Figure 4D).

**FIGURE 5:**
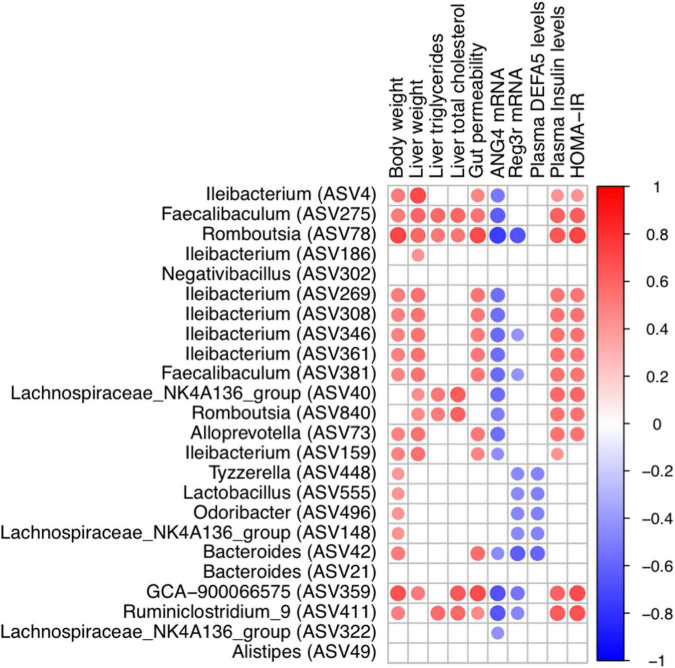
Correlation between metabolic indices and bacterial genera. Heatmap of Spearman’s rank correlation coefficient between metabolic indices and bacterial genera in HC and HD groups was filtered by rho > 0.3 and *p*-value < 0.05. A red line indicates a positive correlation, and a blue line indicates a negative correlation. SC, standard control diet/control; SD, standard control diet/dithizone; HC, high-fat diet/control; HD, high-fat diet/dithizone.

The co-occurrence network in dithizone-treated HFD mice differed from that in non-treated HFD mice, displaying that *Bacteroides ASV21* correlated negatively with *Odoribacter* (*r* = -0.85, *p* = 0.0148) and *Lactobacillus* (*r* = -0.93, *p* = 0.0027) (Figure 4E). *Bacteroides ASV42* was also correlated negatively with the expression of antimicrobial peptides, including ANG4, Reg3γ, and DEFA5 (*r* = -0.45, -0.62, and -0.59, *p* = 0.0116, 0.0002, and 0.0007, Figure 5). The decreases in genera belonging to *Firmicutes* (*Ruminiclostridiumin_9*, *Romboutsia ASV78 and ASV840*, *Faecalibaculum ASV275*, and *Lachnospiraceae_NK4A136_group ASV40*) correlated positively with the corresponding reductions in intrahepatic triglycerides and total cholesterol levels (all *r* > 0.3 and *p* < 0.05, Figure 5).

### The Changes in Microbial Function After Paneth Cell Functional Alterations

**FIGURE 6:**
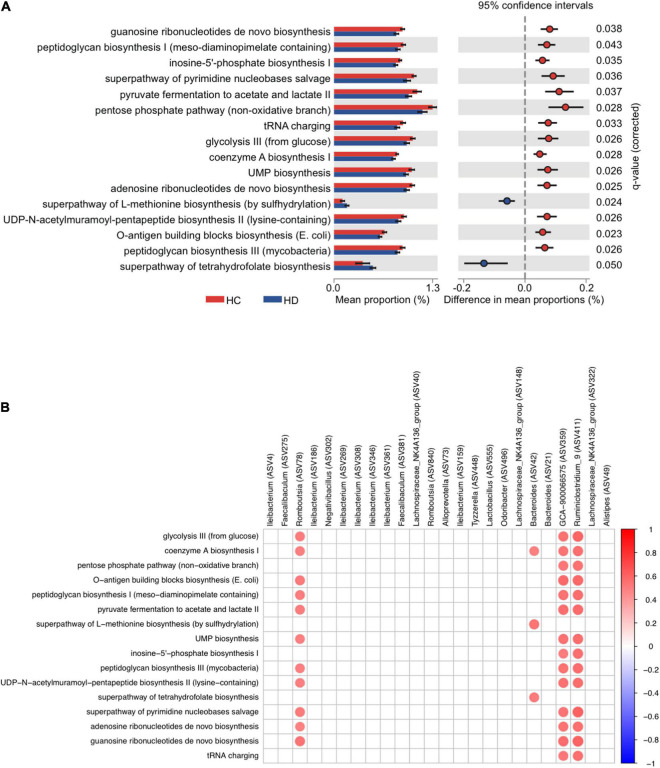
The microbial function of altered microbiota in HFD-fed mice. Mouse fecal microbiome from four groups was analyzed by using PacBio SMRT sequencing (*n* = 7–8 for each group). **(A)** The significantly differential pathways were calculated and visualized with the extended error bar, indicating statistically significant features along with the *p*-values, effect sizes, and confidence intervals. **(B)** Heatmap of Spearman’s rank correlation coefficient between genera and differential pathways was filtered by | Spearman’s rank correlation coefficient| > 0.3 and *p*-value < 0.05. A red dot indicates a positive correlation, and a blue dot indicates a negative correlation. SC, standard control diet/control; SD, standard control diet/dithizone; HC, high-fat diet/control; HD, high-fat diet/dithizone.

Multiple predicted pathways were altered by dithizone treatment in HFD mice. Among them, the superpathways of L-methionine and tetrahydrofolate biosynthesis were significantly enriched (*p* = 0.024 and 0.05, Figure 6A) in dithizone-treated HFD mice when compared to non-treated HFD mice. Correlation analysis demonstrated that *Bacteroides ASV42* had the strongest positive correlation with the superpathway of L-methionine biosynthesis (by sulfhydrylation) (*r* = 0.54, *p* = 0.0019) and superpathway of tetrahydrofolate biosynthesis (*r* = 0.52, *p* = 0.0029) (Figure 6B). In addition, microbiome functional analysis also predicted that the purine/pyrimidine nucleotide biosynthesis-related pathways were significantly decreased in dithizone-treated HFD mice when compared to non-treated HFD mice (all *p* < 0.05, Figure 6A). Purine/pyrimidine nucleotide biosynthesis-related pathways correlated positively with genera belonging to *Firmicutes* (*Romboutsia ASV78*, *Ruminiclostridiumin_9*, and *GCA-900066575*; all *r* > 0.3 and *p* < 0.05, Figure 6B) and also correlated positively with intrahepatic triglycerides and total cholesterol levels (all *r* > 0.3 and *p* < 0.05, Supplementary Figure 5).

## Discussion

**FIGURE 7:**
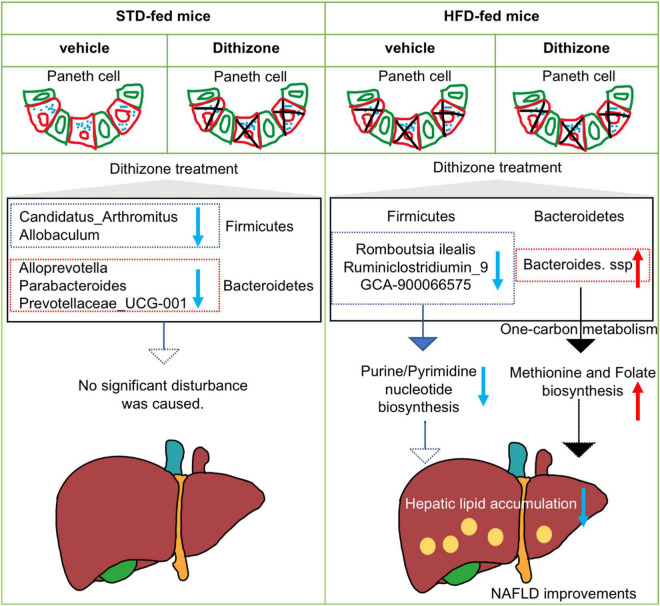
Schematic diagram of the involvement of dithizone-modulated microbial alterations in NAFLD. In STD mice, administration of dithizone significantly disrupted the Paneth cell and altered the microbial composition with no significant disturbance to liver steatosis and glucose metabolism. In HFD mice, dithizone significantly reduced HFD-induced hepatic lipid accumulation. The protective effect of dithizone on NAFLD was mediated by gut microbiome modulation, associated with a notable increase in certain *Bacteroides* species and a decrease in certain *Firmicutes* genera. The presence of *Bacteroides* may result in the upregulated production of L-methionine and folate *via* the superpathway of L-methionine and tetrahydrofolate biosynthesis.

While a great deal of success had been achieved in explaining how gut microbiome dysbiosis leads to NAFLD development, the exact role remains undetermined. The present study had several important findings. First, administration of dithizone significantly disrupted Paneth cells and altered the microbial composition in STD mice; however, no significant disturbance to liver steatosis and glucose metabolism was caused. Second, dithizone significantly reduced HFD-induced hepatic triglycerides and total cholesterol, with improvements in glucose intolerance and insulin resistance and modulatory effects on fatty acid processes. Third, microbial dysbiosis caused by HFD was markedly altered by dithizone, associated with a decrease in the *Firmicutes/Bacteroidetes* ratio and an increase in genus *Bacteroides*. Last, the presence of *Bacteroides* in dithizone-treated HFD mice may result in the upregulated production of L-methionine and folate *via* the superpathway of L-methionine and tetrahydrofolate biosynthesis. Our study findings suggest that dithizone-associated microbial alterations in HFD mice may play a beneficial role in the disease process of NAFLD (Figure 7).

Hepatic steatosis, the major histopathological change with HFD feeding, was alleviated by dithizone treatment. Compared to non-treated HFD mice, dithizone-treated HFD mice had a significantly reduced hepatic triglyceride and total cholesterol content (Figure 2). These results, supported by the modulatory effect of dithizone on lipogenesis and β-oxidation in HFD mice (Supplementary Figure 1), were consistent with previous reports (Postic and Girard, 2008; Xiong et al., 2021).

Paneth cells release their microbicidal granule contents into the gut lumen to maintain gut homeostasis. The function of Paneth cells was disturbed by HFD feeding, evidenced by the decreased ileal lysozyme level and antimicrobial peptides ANG4, Reg3γ, and DEFA5 (Figure 1). Similar results were also noted in STD mice after dithizone administration. Both HFD feeding and dithizone disrupted Paneth cells and downregulated the level of antimicrobial peptides. Recent studies have elucidated that Paneth cells secrete lysozyme *via* secretory autophagy (Bel et al., 2017; Bel and Hooper, 2018; Delorme-Axford and Klionsky, 2018); the disruption of Paneth cells by dithizone administration may also be mediated through an autophagy-related mechanism (Lueschow et al., 2018). Although dithizone treatment resulted in Paneth cell dysfunction in STD mice, no significant disturbance to markers of liver steatosis was caused (Figure 2), and the altered genus induced by dithizone administration (Supplementary Figure 4A) significantly differed from that induced by HFD feeding (Supplementary Figure 4B), suggesting that Paneth cell dysfunction *per se* is not the causal factor contributing to NAFLD development.

The beneficial effects of dithizone only become relevant in an HFD environment. In the normal condition, the effects of dithizone on Paneth cells, which downregulate the levels of antimicrobial peptides (Figure 1), are considered as a kind of side effects; no other additional effects on liver function were observed. However, the side effect of dithizone is not prominent in an HFD environment, since HFD feeding also disrupted the function of Paneth cells. In this case, besides dithizone functioning as an inhibitor of antimicrobial peptides, our results showed that dithizone treatment alleviates the severity of liver steatosis by modulating the gut microbiome in HFD mice.

An increase in the ratio of two domain phyla *Firmicutes* and *Bacteroidetes* has been widely considered a signature of gut dysbiosis and associated with NAFLD (Ley et al., 2006; Porras et al., 2017). In the current study, dithizone treatment led to a decreased *Firmicutes*/*Bacteroidetes* ratio in HFD mice. Moreover, *Bacteroides* presented in dithizone-treated HFD mice with higher abundance than in non-treated HFD mice. However, in STD mice, no significant difference was observed in the abundance of genus *Bacteroides* between non-treated and dithizone-treated STD mice. Whether dithizone had a direct effect on promoting the growth of *Bacteroides* species needs to be further determined by an *in vitro* growth curve study. Prior studies have shown that several *Bacteroides* species alleviate lipid metabolic disorders; *Bacteroides thetaiotaomicron*, *vulgatus*, and *acidifaciens* were associated with reductions in diet-induced body weight gain, attenuations in atherosclerosis, and improvements in insulin sensitivity, respectively (Liu et al., 2017; Yang et al., 2017; Yoshida et al., 2018). Meanwhile, *Ruminiclostridiumin_9*, *Romboutsia*, and *GCA-900066575*, all microbes belonging to *Firmicutes* and with associations to obesity (Gao et al., 2020; Horne et al., 2020), were decreased in dithizone-treated HFD mice when compared to non-treated HFD mice. *Romboutsia ASV78* was further annotated as *Romboutsia ilealis* (matched with 100% identity by NCBI BLAST); a recently published study has demonstrated that supplementation with *Romboutsia ilealis* in mice resulted in glucose intolerance (Rodrigues et al., 2021).

One-carbon metabolism comprises interrelated methionine and folate pathways (Clare et al., 2019). Folate has the beneficial effects of alleviating hepatic steatosis (Qiao et al., 2020), while its depletion may cause oxidative stress in the liver and lead to the development of NAFLD (Huang et al., 2001). Methionine is an essential amino acid involved in DNA methylation, with its depletion leading to liver steatosis in mice (Pacana et al., 2015). Indeed, a methionine- and choline-deficient diet is usually used to establish an animal NAFLD model covering the whole spectrum of liver pathologies from steatosis to hepatic fibrosis (Stephenson et al., 2018). In our study, we demonstrated that one species from the genus *Bacteroides*, ASV42 (matched to *Bacteroides vulgatus* with 99.931% identity by NCBI BLAST), had a positive relationship with producing folate and L-methionine. However, the intervention study and the mechanistic insights of *Bacteroides* species and its metabolites folate and L-methionine in the prevention of NAFLD development will need further confirmation.

Our study findings would lead to an intriguing clinical question: can therapies aimed at these microbial alterations be effective in treating NAFLD? This question is especially relevant given the paucity of pharmaceutical options currently available for the disease. Microbiome-based therapies targeting different *Bacteroides* species may be a possible approach. That being said, our study is limited by the inadequate microbial classification of *Bacteroides*. The two *Bacteroides* species found in this study would require further classification *via* full-length sequencing, as well as microbial isolation and identification. Moreover, further animal experiments are required to investigate whether Paneth cell dysfunction-related microbial changes are the causal factor contributing to NAFLD development by colonizing mice with fecal microbiota from dithizone-treated mice. Other limitations include the lack of analysis for actual microbiome functional pathways which may provide better mechanistic insight on Paneth cell dysfunction and NAFLD and the lack of tissue metabolomic data to further clarify the role of bacteria-fermented metabolites in the development of NAFLD.

In summary, dithizone alleviated the severity of NAFLD, manifesting as a decrease in intrahepatic lipid accumulation, accompanying improvements in glucose intolerance and insulin resistance. The protective effect of dithizone on NAFLD was mediated by gut microbiome modulation, potentially associated with the notable increase in *Bacteroides* species and decrease in some *Firmicutes* genera. Our study strengthens the understanding of gut-related mechanisms in NAFLD development. Future research will be needed to study if *Bacteroides* species can emerge as a novel therapeutic option for NAFLD.

## Data Availability Statement

The datasets presented in this study can be found in online repositories. The names of the repository/repositories and accession number(s) can be found in the article/Supplementary Material.

## Ethics Statement

The animal study was reviewed and approved by the Committee on the Use of Live Animals in Teaching and Research at the University of Hong Kong (reference number: CULATR 4507-17).

## Author Contributions

SZ was involved in study concept and design, laboratory measurements, acquisition of data, analysis, interpretation of data, and drafting of the manuscript. HT, DZ, H-TC, F-YH, HK, DW, and L-YM were involved in the acquisition of data, analysis, and interpretation of data. M-FY was involved in the study concept and design and critical revision of the manuscript. W-KS was involved in study concept and design, securing research funding, analysis and interpretation of data, critical revision of the manuscript, and overall study supervision. All authors declare that they have participated in the preparation of the manuscript and have seen and approved the final version.

## Conflict of Interest

M-FY is an advisory board member and/or received research funding from AbbVie, Arbutus Biopharma, Assembly Biosciences, Bristol Myers Squibb, Dicerna Pharmaceuticals, GlaxoSmithKline, Gilead Sciences, Janssen, Merck Sharp and Dohme, Clear B Therapeutics, Springbank Pharmaceuticals; and received research funding from Arrowhead Pharmaceuticals, Fujirebio Incorporation, and Sysmex Corporation. W-KS received speaker’s fees from AstraZeneca and Mylan, is an advisory board member of CSL Behring, is an advisory board member and received speaker’s fees from AbbVie, and is an advisory board member, received speaker’s fees and researching funding from Gilead Sciences. The remaining authors declare that the research was conducted in the absence of any commercial or financial relationships that could be construed as a potential conflict of interest.

## Publisher’s Note

All claims expressed in this article are solely those of the authors and do not necessarily represent those of their affiliated organizations, or those of the publisher, the editors and the reviewers. Any product that may be evaluated in this article, or claim that may be made by its manufacturer, is not guaranteed or endorsed by the publisher.

## Footnotes

1
https://CRAN.R-project.org/package=vegan
