# Supplementary material for: Alleviation of Hepatic Steatosis: Dithizone-Related Gut Microbiome Restoration During Paneth Cell Dysfunction
Source: Front Microbiol. 2022 Feb 25;13:813783. doi: 10.3389/fmicb.2022.813783 (PMC8914291; doi:10.3389/fmicb.2022.813783)
Supplement: Supplementary file 1 [file Data_Sheet_1.docx]

**Alleviation of hepatic steatosis: dithizone-related gut microbiome restoration during Paneth cell dysfunction**

Saisai Zhang, Hein M Tun, Dengwei Zhang, Hau-Tak Chau, Fung-Yu Huang, Hin Kwok, Danny Ka-Ho Wong, Lung-Yi Mak, Man-Fung Yuen, Wai-Kay Seto*

**Supplementary Material**

**
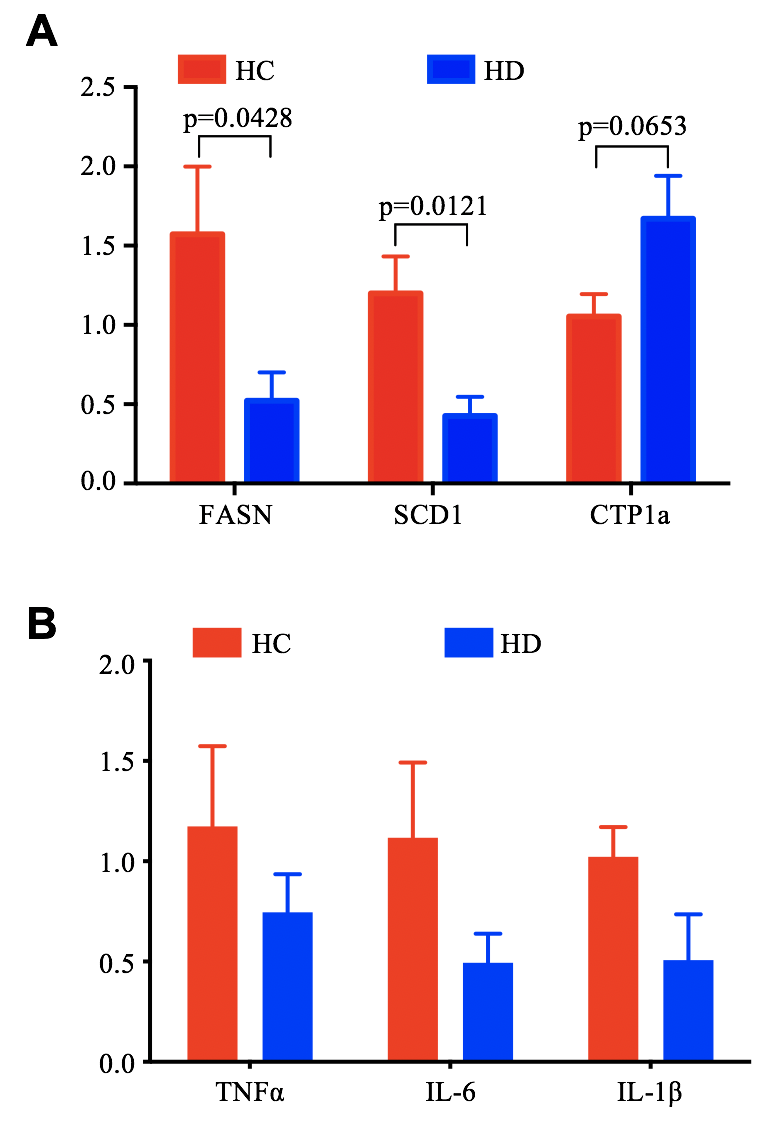
Figure Legends**

**Supplementary Figure S1. The regulating role of dithizone in fatty acid synthesis, β-oxidation in HFD mice.** The relative mRNA levels of fatty acid synthesis-related genes FASN and SCD1, and β-oxidation related gene CTP1a.

SC, standard control diet/control; SD, standard control diet/dithizone; HC, high-fat diet/control; HD, high-fat diet/dithizone; SEM, standard error of the mean; FASN, fatty acid synthase; SCD1, Stearoyl-CoA desaturase-1; CTP1a, Carnitine palmitoyltransferase 1a.

**Supplementary Figure S2. The effect of dithizone on the intestinal barrier.** (**A**) Representative images of ileum stained with H&E with x400 magnification. (**B**) Total gut permeability by fluorescein isothiocyanate (FITC)-dextran assay. **(C)**The protein expression of zonula occludens (ZO)-1 and occludin were determined by western blot analysis (upper); Densitometric quantification was calculated by ImageJ software. Data are expressed as mean ± SEM for each group.

SC, standard control diet/control; SD, standard control diet/dithizone; HC, high-fat diet/control; HD, high-fat diet/dithizone; SEM, standard error of the mean

**Supplementary Figure S3. Fecal microbial diversity.** (**A**) Chao 1 (a measure of community richness) and Shannon’s index (a measure of abundance and evenness). (**B**) Principal coordinates analysis (PCoA) of microbiota based on Unweighted UniFrac Distance (a qualitative measure of community dissimilarity) was adopted. The comparison was tested by the Wilcoxon rank-sum test. P-value was corrected using the Benjamini–Hochberg method, named as false discovery rate (FDR) value.

**
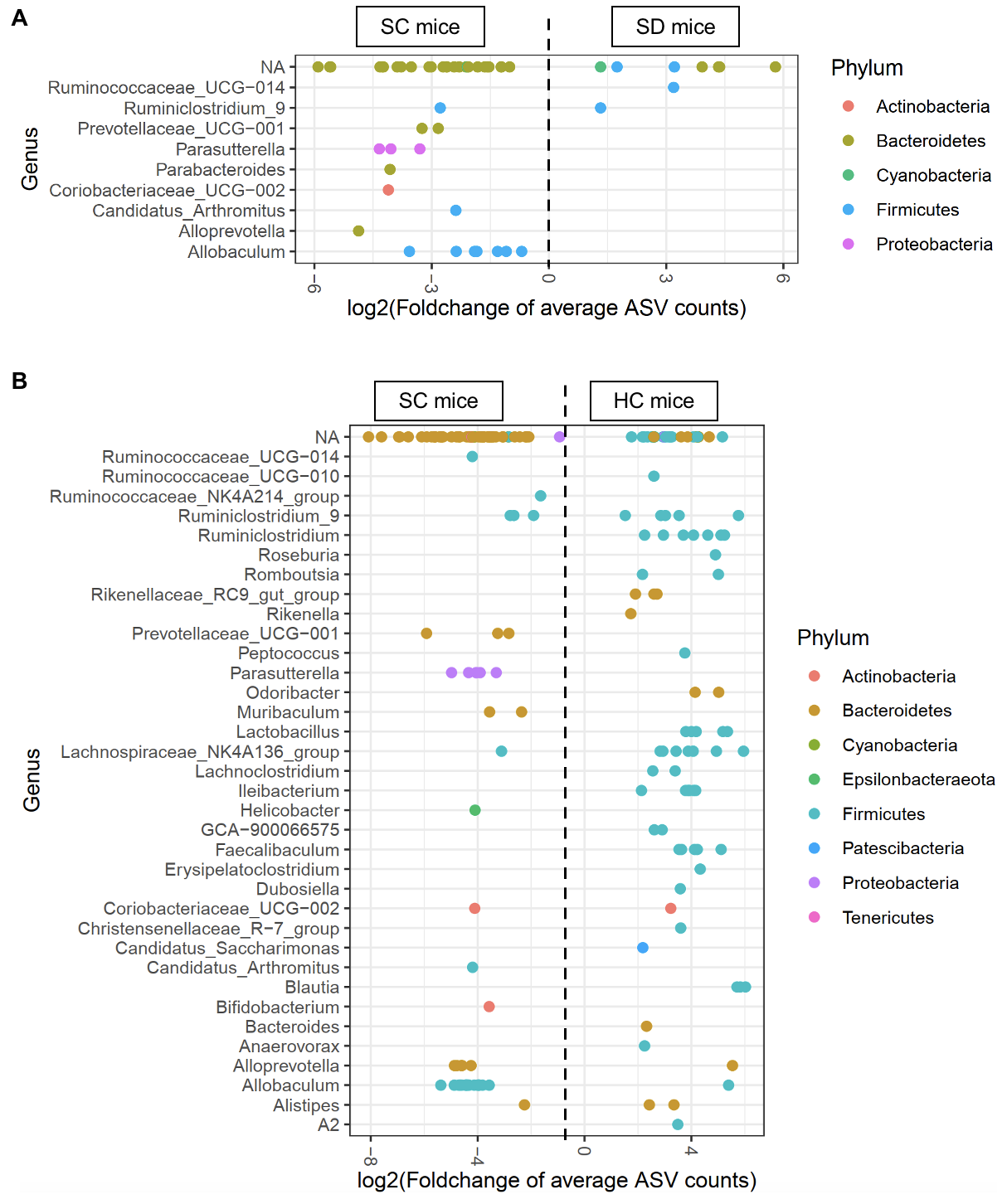
**

**Supplementary FigureS4.** The altered bacterial genera in (**A**) non-treated HFD mice and (**B**) dithizone-treated STD mice when compared to non-treated STD mice.

SC, standard control diet/control; SD, standard control diet/dithizone; HC, high-fat diet/control.

**
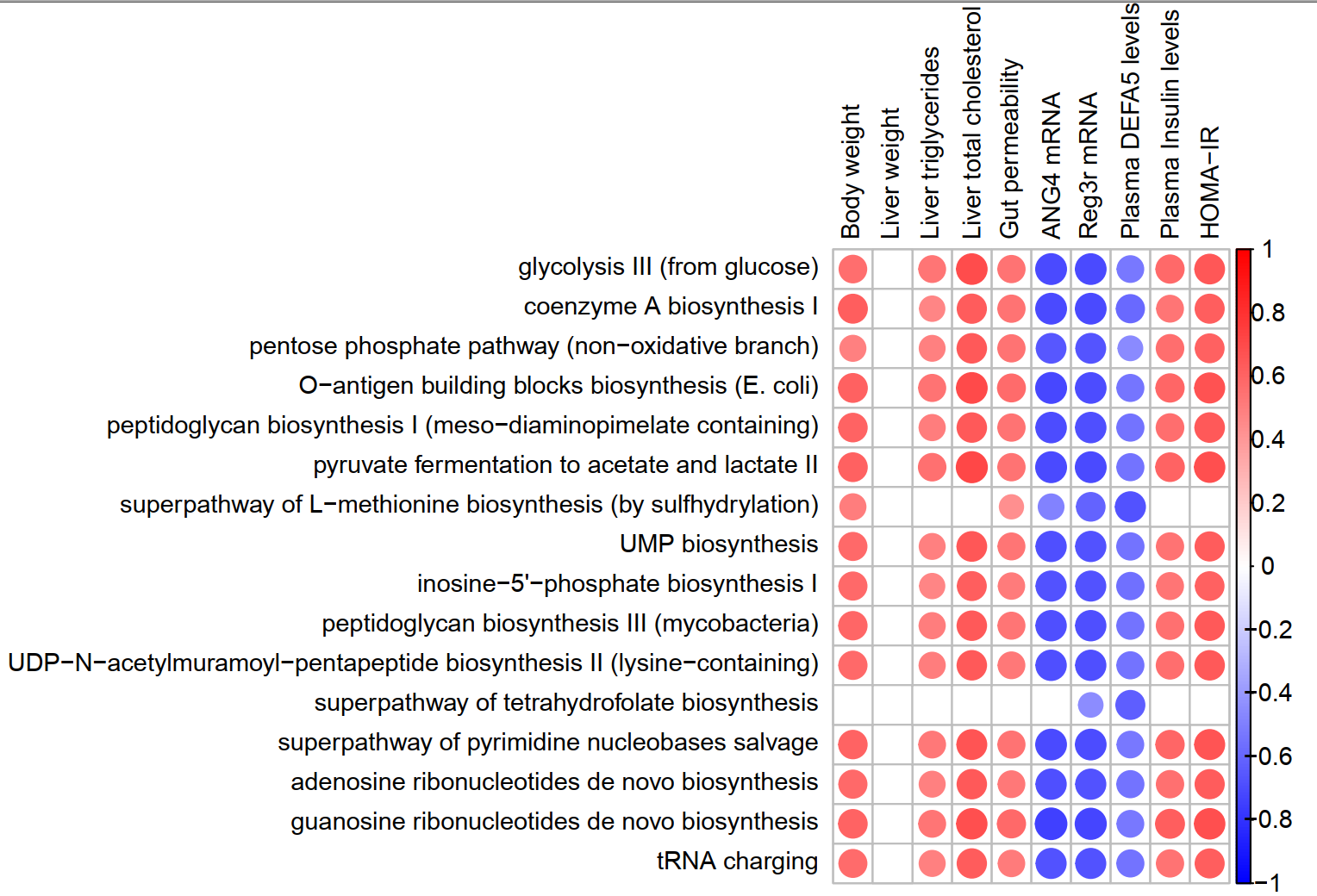
**

**Supplementary Figure S5.** Heatmap of the Spearman’s rank correlation coefficient between metabolic indices and differential pathway in HC and HD groups was filtered by |Spearman’s rank correlation coefficient| > 0.3 and p-value < 0.05. The red dot indicated a positive correlation and the blue dot indicated a negative correlation.

**Supplementary Table S1. The sequences of primers.**

| Gene |  | Sequence (5’-3’) |
| --- | --- | --- |
| ANG4 | Forward  Reverse | \| GGTTGTGATTCCTCCAACTCTG \| \| --- \| \| CTGAAGTTTTCTCCATAAGGGCT \| |
| Reg3γ | Forward  Reverse | \| ATGCTTCCCCGTATAACCATCA \| \| --- \| \| GGCCATATCTGCATCATACCAG \| |
| FASN | Forward  Reverse | \| GGAGGTGGTGATAGCCGGTAT \| \| --- \| \| TGGGTAATCCATAGAGCCCAG \| |
| SCD1 | Forward  Reverse | \| TTCTTGCGATACACTCTGGTGC \| \| --- \| \| CGGGATTGAATGTTCTTGTCGT \| |
| CTP1a | Forward  Reverse | \| CTCCGCCTGAGCCATGAAG \| \| --- \| \| CACCAGTGATGATGCCATTCT \| |
| β-actin | Forward  Reverse | \| GGCTGTATTCCCCTCCATCG \| \| --- \| \| CCAGTTGGTAACAATGCCATGT \| |

ANG4, angiogenin 4; Reg3γ, regenerating islet-derived protein 3 gamma; FASN, fatty acid synthase; SCD1, stearoyl-CoA desaturase-1; CTP1a, carnitine palmitoyltransferase 1a.
